# Recombinant *Escherichia coli* BL21-pET28a-*egfp* Cultivated with Nanomaterials in a Modified Microchannel for Biofilm Formation

**DOI:** 10.3390/ijms19092590

**Published:** 2018-08-31

**Authors:** Chang-Tong Zhu, Yi-Yuan Mei, Lin-Lin Zhu, Yan Xu, Sheng Sheng, Jun Wang

**Affiliations:** 1 School of Biotechnology & School of the Environment and Chemical Engineering, Jiangsu University of Science and Technology, Zhenjiang 212018, China; zct2033@sina.com (C.-T.Z.); myy876323459@sina.com (Y.-Y.M.); zhangxuexuan12@126.com (L.-L.Z.); xuyan@just.edu.cn (Y.X.); shengsheng@just.edu.cn (S.S.); 2 Sericulture Research Institute, Chinese Academy of Agricultural Sciences, Zhenjiang 212018, China

**Keywords:** biofilm, microreactor, surface modification, nanomaterials, recombinant *Escherichia coil*

## Abstract

The application of whole cells as catalytic biofilms in microchannels has attracted increasing scientific interest. However, the excessive biomass formation and structure of biofilms in a reactor limits their use. A microchannel reactor with surface modification was used to colonize recombinant *Escherichia coil* BL21-pET28a-*egfp* rapidly and accelerated growth of biofilms in the microchannel. The segmented flow system of ‘air/culture medium containing nanomaterials’ was firstly used to modulate the biofilms formation of recombinant *E. coil*; the inhibitory effects of nanomaterials on biofilm formation were investigated. The results indicated that the segmental flow mode has a significant impact on the structure and development of biofilms. Using the channels modified by silane reagent, the culture time of biofilms (30 h) was reduced by 6 h compared to unmodified channels. With the addition of graphene sheets (10 mg/L) in Luria-Bertani (LB) medium, the graphene sheets possessed a minimum inhibition rate of 3.23% against recombinant *E. coil*. The biofilms cultivated by the LB medium with added graphene sheets were stably formed in 20 h; the formation time was 33.33% shorter than that by LB medium without graphene. The developed method provides an efficient and simple approach for rapid preparation of catalytic biofilms in microchannel reactors.

## 1. Introduction

Microorganisms mainly exist in nature in the form of biofilms [1]. Biofilms are mainly composed of surface-related microorganisms and flocs or aggregates formed by microorganisms, which can be adapted to various environments. Biofilms can be formed at a wide range of interfaces, such as oil/water/air [2,3]. Biofilms embed their own secreted extracellular polymers, which are beneficial to the adaptation of growth environment. As a new type of whole-cell catalyst for chemical synthesis, biofilms have been used in the wider fields of fine chemicals, biofuels, and microbial fuel cells [4,5]. However, in a batch reactor, microbial cells are often grown suspended in a liquid medium and cannot be reused. This results in the limitation of mass transfer, which reduces the working efficiency of the reactor [6]. In order to solve the problem of uncontrollable formation of biofilms, it is urgent to find a method for inhibiting the growth of biofilms.

Microfluidic reaction devices are a very promising technology for biochemical processes. In microfluidics, efficient heat transfer, narrow residence time distribution, rapid system response, and easy automatic control can result in higher reaction yields and reaction rates than in conventional reactors [7,8]. Microscale channels are closer to the biological environment and microfluidics can precisely control the cells in a particular growth environment. Biofilms have been applied in microreactors [9,10]. The highly cationic chemically modified plasma protein bovine serum albumin cBSA-147 has been successfully used as a biocompatible adhesion promoter for the biofilm formation of recombinant *Escherichia coli* whole cells in a capillary column [11]. Although the immobilization process of recombinant *E. coli* whole cells occurred very rapidly under mild conditions, the mass transfer and reactor blocking will be restricted because of the excessive biomass formation in microchannel reactors. Aqueous-air segmented flow has been introduced into microreactors, which could effectively control excessive biofilm growth. Excellent works have also been done for biofilm colonization and the growth of catalytic biofilms could be controlled by aqueous-air segmented flow in continuous microreactors [12]. With this method, the epoxidation of styrene to (*S*)-styrene oxide (e.e. > 99.8%) was catalyzed by *Pseudomonas* sp. strain VLB120DC cells in the biofilms.

In the process of biofilm formation, many factors affect the adhesion of bacteria to the surface: the availability of surface nutrients, nutrient concentration, pH value of the growth environment, temperature, electrolyte concentration, adhesion matrix material, specific surface, etc. [13]. Thus, the growth of a biofilm is affected by initial cell adhesion in the microchannels. Surface properties of microchannels play an important role in initial cell attachment. Modifications such as the use of appropriate coatings or a rough surface have been suggested to promote cell–surface interactions and provide protection from shear forces, and hence encourage cell settlement and biofilm growth [14]. Meanwhile, for catalytic biofilms in microreactors, the biofilm structure will affect the catalytic efficiency of biofilms. Especially, for the catalytic biofilms of recombination strains expressing enzymes in the cells, more biomass was difficult for the mass transfer of the substrate in the liquid phase. Therefore, surface modification of the microchannels and improvement of the biofilm structure are important for enhancing biofilm growth and catalytic efficiency.

In addition, aminosilanes and bifunctional cross-linkers are commonly used for surface modification for biocatalyst immobilization. 3-aminopropyl-triethoxysilane (APTES) has been used for the introduction of amino-groups, further reacting with glutaraldehyde, which form covalent bonds with primary amines on the surfaces of enzymes or cells [15,16]. By this method, *E. coli* covered approximately 67% on the functionalization of perfluoroalkoxy (PFA) microchannels. Therefore, it is feasible to accelerate the growth of a bacterial biofilm by accelerating the adsorption rate of the strain onto the surface of the microchannel. Nanoscale materials have recently appeared as one of the most promising strategies to control biofilms. These nanomaterials are made from different metals (copper, zinc, titanium, magnesium, gold, silver, etc.) and their salts or polymers. The silver nanoparticles (AgNP) were effective against Gram-negative bacteria resistant to antibiotics, and strongly inhibited biofilms. The strong antibacterial action of nanomaterials are not suitable for improving the structure of biofilms. Carbon nanomaterials, as a new type of highly efficient adsorption carrier, have the advantages of larger specific surface area, higher loading capacity, ordered nanopore structure, excellent chemical stability, and biocompatibility [17]. Carbon nanomaterials such as carbon nanotubes and graphene have attracted widespread attention in the field of biocatalysis [18]. Multiwalled carbon nanotubes were used for the immobilization of lipase in the conversion of Jatropha oil to fatty acid methyl esters, which showed excellent adsorption capacity and biocompatibility [19]. Thus, the carbon nanomaterials embedded in biofilms have the potential of solving the problems of catalytic biofilm structure.

In the present study, a silane agent was used to modify microreactor surface for rapid colonization of recombinant *E. coli* BL21-pET28a-*egfp* and Luria-Bertani (LB) medium containing nanomaterials was used to cultivate biofilms by segmentation flow. The impacts of surface modification and nanomaterials on the formation and structure of biofilms in a microreactor were investigated. This method is highly promising in the application of catalytic biofilms of recombination strains expressing enzymes in microreactors.

## 2. Results and Discussion

### 2.1. Biofilm Growth Under Segmented Flow and Single Phase Flow Modes

**Figure 1:**
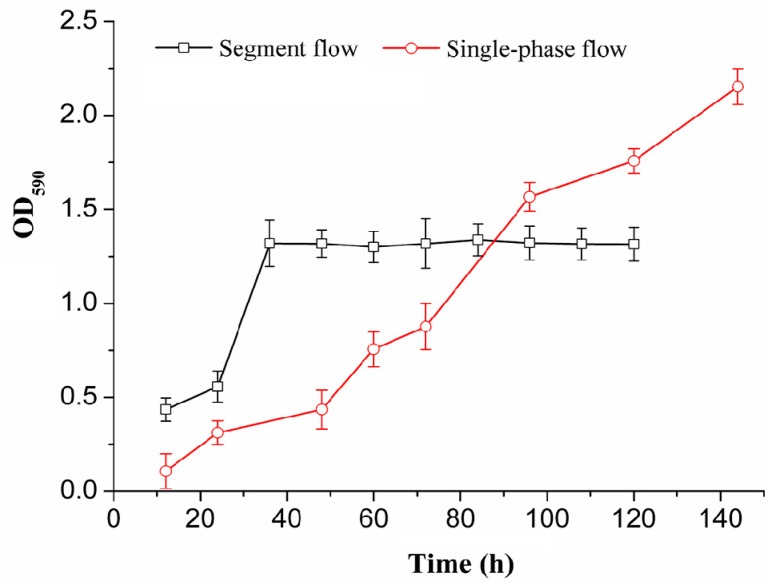
Growth of recombinant *E. coli* BL21-pET28a-*egfp* biofilms in a microchannel reactor under different flow modes.

Figure 1 shows the difference between the total biomass of recombinant *E. coli* BL21-pET28a-*egfp* biofilms cultivated by segmented flow and single phase flow. Under the segmented-flow mode, the total amount of biofilms in the microchannels had been increasing before 36 h and the growth rate of biofilms was rapid between 24 and 36 h. After 36 h, the amount of biofilms remained constant. Although the total amount of biofilms was not changed, the recombinant *E. coli* were constantly washed out from the channel. Under the single-phase flow mode, the growth rate of biofilms emerged to be slow by measuring the total amount of biofilms. However, the total biomass of biofilms was increased sharply after 36 h. The total biomass of biofilms cultivated by single-phase flow was less than the growth of the segmented flow before 80 h. After the stability period of biofilms growth by segmented flow, the biofilms cultivated by single-phase flow could continue to grow, which was consistent with previous results [20]. Comparing the total biomass of *E. coli* biofilms grown in different fluid flow, the growth rate of biofilms under segmented flow mode was higher than that under the single-phase flow mode before 36 h. The results showed that the biofilms growth in the microchannels under the segmented flow mode had been effectively controlled. This is because the internal convection of the aqueous segments enhanced the mass transfer arising from the segmented flow, which was beneficial to increase the growth rate of the biofilms. In addition, the oxygen input in the air segments was beneficial to increase the growth rate of biofilms [21]. Thus, fluid flow regimes have a significant impact on the structures and development of biofilms.

### 2.2. Biofilm Cultivation in the Microchannel Reactor

**Figure 2:**
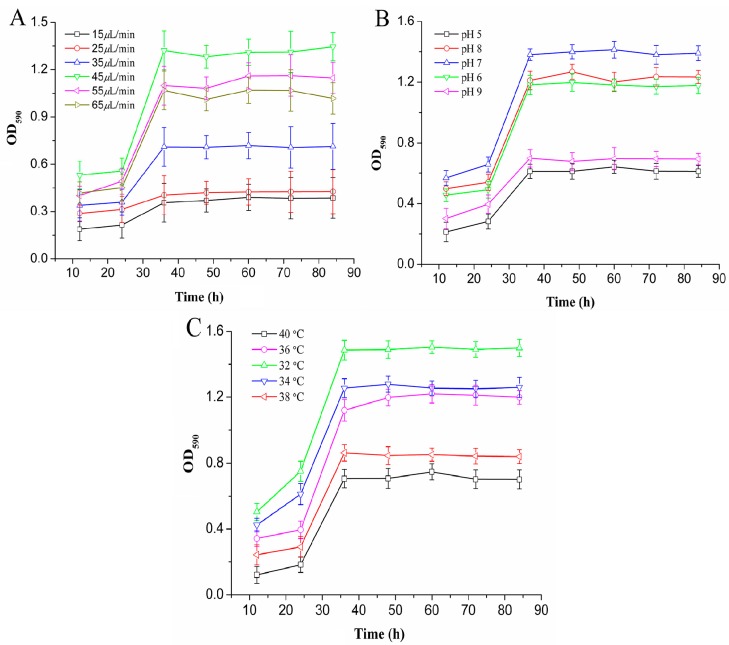
Effects of different flow rates (**A**), pH (**B**), and temperature (**C**) on the formation of recombinant *E. coli* BL21-pET28a-*egfp* biofilms.

Figure 2A shows the effect of the flow rate on the total amount of biofilms. It was observed that the growth rate of the biofilms was the fastest and the total biomass was the highest, when the flow rate was 45 μL/min. The flow rate and the concentration of nutrient solution were two important factors affecting the structure of the biofilms in the microchannel. At a flow rate between 15 and 35 μL/min, the total amount of biofilms increased. This was because that the increasing flow rate could enhance the mass transfer within the biofilms under the constant concentration of nutrient solution, thus significantly increasing the total amount of biofilms. When the flow rate exceeded 45 μL/min, the total biomass started to decrease with the increase of flow rate. This is because the flow rate increases the fluid shear stress at the same time as the mass transfer [22]. Increased fluid shear forces could lead to biofilm detachment from the surface of the microchannels and affect the physical properties of biofilms, thereby reducing the total biological population [23]. When the microchannel surface properties, nutrient concentration, pH, and temperature of the growth environment are constant, the flow rate determined the growth rate and total biomass of the biofilms. Therefore, 45 μL/min was selected as the appropriate flow rate.

As shown in Figure 2B, the growth period of the biofilms was relatively insignificant, and the total biomass of biofilms in the mature period was low at pH values of 5 or 9. This was due to the effect of environmental pH value on the growth of the recombinant *E. coli* biofilms. A peracid environment was not suitable for the formation of biofilms in microchannels [24]. In the pH range of 6 to 8, the growth of the biofilms in the microchannel showed obvious adhesion, growth, and maturation phases, and the growth rate of the biofilms was relatively fast. At pH 7, the biofilms developed rapidly with the largest amount of biomass. Therefore, pH 7 was the appropriate environmental parameter for the biofilm formation in the microchannel reactor.

In addition, temperature is an important factor to influence the growth rate and metabolism of bacteria. The biofilm formation was affected by culture temperature [25]. Figure 2C shows that the total biomass of the recombinant *E. coli* biofilms in the microchannel was higher than that at others, when the culture temperature was 32 °C and 34 °C. The total amount of biofilms reached its maximum value at 32 °C. Compared with the conventional method of culturing recombinant *E. coli*, the temperature reduced, due to the better heat transfer in the microchannel reactor. At 38–40 °C, the total biomass of biofilms formed by *E. coli* in microchannels was relatively low. This was due to the fact that the metabolic heat production of bacteria was difficult to transmit in high temperature environments, which hindered the normal living activity of cells. A decrease of the concentration of bacteria on the microchannel wall and the growth of the biofilms was reduced. Therefore, 32 °C was chosen as a suitable growth temperature for the cultivation of biofilms in the microchannel reactor.

Table 1 shows the growth of biofilms at different oxygen content. The oxygen content positively changed with the air flow rate. When the air flow rate was 95 μL/min, which was 2.1-fold greater than the aqueous flow rate, the total biomass of biofilms increased by 1.1-fold. Due to the increased flow rate, the mass transfer rate of nutrients was increased. At the same time, the content of oxygen dissolved in the aqueous phase increased, which was also conducive to accelerating the growth of biofilms [12]. However, the total biomass of biofilms remained unchanged at the rate of 180 μL/min. Essentially, enough oxygen, which was saturated in the microchannel, supported the growth of biofilms in the system. In addition, when the flow rate was too high, the residence time of nutrients in the microchannel reactor decreased, resulting in a decrease in the growth rate of biofilms. Therefore, a suitable air flow rate was conducive to biofilm formation.

### 2.3. Effect of Channel Surface Modification on Cell Immobilization and Biofilm Formation

**Figure 3:**
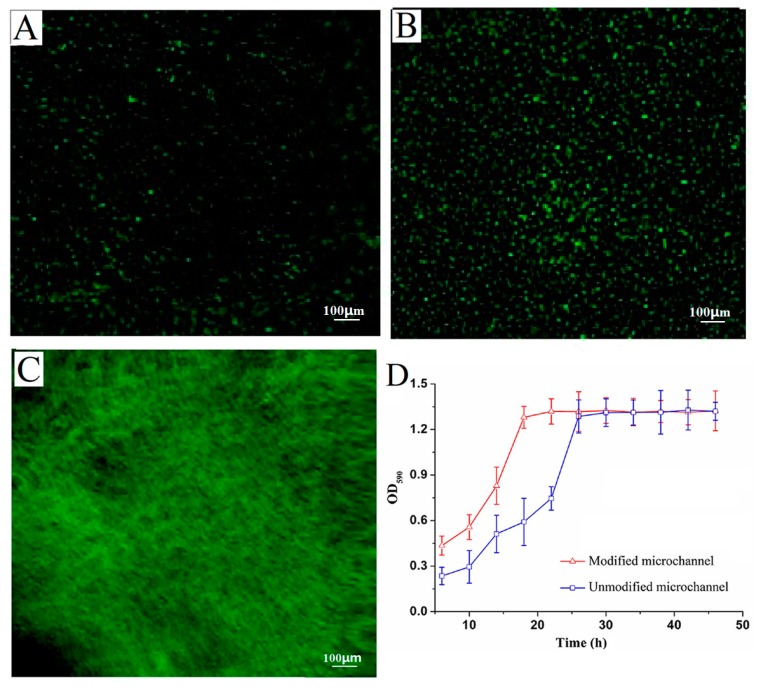
The photos of recombinant *E. coli* BL21-pET28a-*egfp* on unmodified (**A**) and modified (**B**) microchannel surfaces under inverted fluorescence microscope. The photo of recombinant *E. coli* biofilms on the modified microchannel surfaces under inverted fluorescence microscope after 30 h (**C**). The total biomass of recombinant *E. coli* biofilms were compared in unmodified and modified microchannels (**D**).

The morphology of the recombination *E. coli* adhered onto the surface of microchannel was studied using fluorescence inversion microscope (Figure 3A,B).

With the surface of microchannel modified by 3-aminopropyltriethoxysilane (ATPES), the strain adsorption capacity at the same time was different in a microchannel reactor. Figure 3A shows slight green fluorescence at the unmodified channel surface after 2 h, which suggested the recombination *E. coli* strains were absorbed onto the surface and began to grow. As shown in Figure 3B, much more green fluorescence was observed at the modified channel surface after 2 h. The results indicated that much more strains were absorbed onto the modified surface and grew well. The result was due to the introduction of amino-groups from APTES, which further reacted with glutaraldehyde forming covalent bonds with primary amines from the cells on the channel surfaces [26]. Moreover, the high-density growing biofilms were in homogeneous distribution on the modified channel surface (Figure 3C). On the whole, Figure 3D shows that the recombinant *E. coli* biofilms were cultivated at 46 h in unmodified and modified microchannel reactors. In the microchannels unmodified and modified by the silane reagent, the total amount of biofilms reached the maximum within 36 h and 30 h, respectively. Thus, the culture time of recombination *E. coli* biofilms was reduced by 6 h in the modified channel reactor.

The results indicated that a large number of strains adhered on the surface of the modified microchannel were more stable in the initial stage of biofilm growth. This stability resulted from the stronger resistance to fluid shear force caused by the increase of adhesion strength, thereby, accelerating the growth rate of the biofilms [27]. However, the growth of the biofilms was limited by factors such as flow rate, oxygen content, and nutritional status; the total amount of the biofilms eventually reached a steady value with a tendency to balance [28]. There was no significant difference in the total biomass of biofilms in modified and unmodified microchannels after equilibrium was reached. That might be due to the fact that the growth of the biofilms in the segmented flow microchannel was only related to the fluid flow in the channel and oxygen consumption. It had nothing to do with the amount of strain initially adsorbed, but the initial amount of bacteria affected the rate of growth of the biofilms. Therefore, the surface modification of channels was used to adsorb the recombinant strains which then had a positive effect on shortening the culture time of the biofilms.

### 2.4. Biofilm Cultivation with Nanomaterials

**Figure 4:**
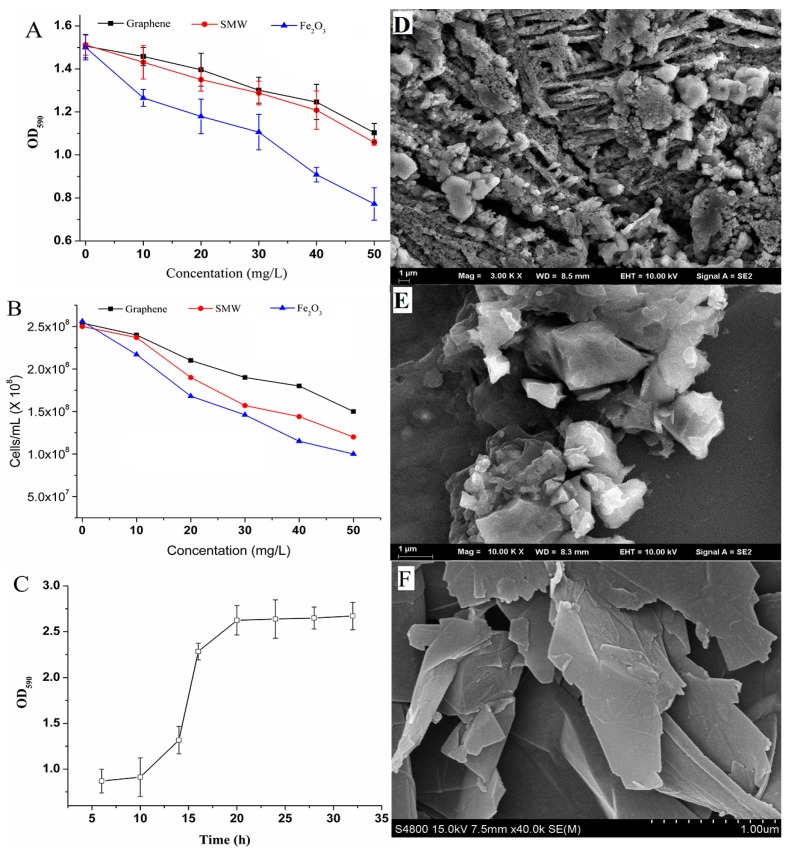
Effects of different nanomaterials and concentrations on growth of biofilms (**A**) and recombinant *E. coli* BL21-pET28a-*egfp* (**B**). The growth of recombinant *E. coli* biofilms with graphene nanomaterial was measured under segment flow mode (**C**). The SEM patterns of biofilms formed in microchannels with LB medium (**D**), LB medium containing graphene sheets (10 mg/L) (**E**), and graphene sheets (**F**).

As shown in Figure 4A, when the concentration was 10 mg/L, the inhibition rate of graphene sheets on the biofilm formation was only 3.23% compared with the blank group. At a concentration of 50 mg/L, the growth inhibition rate of graphene on the biofilms increased to 26.76%. When the concentration of carbon nanotubes was 10 mg/L and 50 mg/L, their growth inhibition rates on biofilms were 7.51% and 29.74%, respectively. Fe_2_O_3_ nanoparticles had the greatest effect on biofilm formation. Its growth inhibition rate on biofilms was as high as 16.13% and 48.53%, with concentrations of 10 mg/L and 50 mg/L, respectively. Compared with carbon nanotubes and Fe_2_O_3_, the inhibition rate of graphene on the total biomass of biofilms was reduced by 56.99% and 79.97%, respectively, when the concentration was 10 mg/L.

Figure 4B shows that the nanomaterials have varying degrees of inhibitory effect on the growth of recombinant *E. coli*. With a low concentration of nanomaterials, the growth inhibition on the recombinant *E. coli* was weak. With the concentration reaching 50 mg/L, nanomaterials showed significant inhibitory behavior on the recombinant *E. coli* growth. The inhibitory effect of graphene on the recombinant *E. coli* was relatively weak. When graphene concentrations were 10 mg/L and 50 mg/L, the relative inhibition rates were 5.59% and 40.94%, respectively. Fe_2_O_3_ nanoparticles showed the greatest inhibitory effect on the recombinant *E. coli*, and inhibited the cell growth slightly more than carbon nanotubes. At concentrations of 10 mg/L and 50 mg/L, the carbon nanotubes and Fe_2_O_3_ nanoparticles inhibition on the recombinant *E. coli* were 13.72%, 48.56%, 17.98%, and 60.93%, respectively. Compared with the inhibition results of biofilm growth, the inhibitory effect of nanomaterials on biofilms was less than that of recombinant *E. coli* at the same concentration. When the concentration was 10 mg/L, the inhibition rate of graphene on biofilms was lower, which decreased by 42.21%.

The result suggested that the recombinant strains in mature biofilms are less sensitive to the presence of nanomaterials. It was because of the extracellular polymeric substances (EPS) secreted from the cell surface contributed to protecting mature biofilms, and mitigating the inhibition effects of nanoparticles inside biofilms [29]. The extracellular polymer surrounded by the biofilms can hinder the penetration of the nanomaterial into the cell and adsorb a part of the nanomaterial, which increases the tolerance of the biofilms to the nanomaterial and reduces the inhibition effect [30,31].

Figure 4C shows the effect of graphene on the biofilm growth curve in the segmented flow microreactor. After being cultured for 10 h, the increase in the rate of total biomass of biofilms was relatively low which later increased the total amount of biofilms rapidly. When the culture time reached 20 h, the total amount of biofilms reached its maximum and remained unchanged. Compared to the culture results without nanomaterials in Figure 3D, the culture time was reduced by 10 h. This was because the graphene sheets adsorbed on the microchannel suffice and embed the biofilms which adsorbed large amount of proteins from the LB medium, and thus the proteins could provide sufficient nutrients for recombinant strain growth and biofilm formation [32]. Thus, the graphene sheets wrapped in biofilms stabilized the biofilm structure and accelerated the growth of biofilms.

In summary, the above results were mostly based on the special physicochemical properties of nanomaterials such as microsize, large specific surface area, and high surface activity. Table 2 shows the differences of nanomaterials physical parameters. The larger density of nanomaterials could deposit onto the bottom, which led to a high concentration of nanomaterials in biofilms and increased the inhibition effects for biofilm formation, while flowing in the microchannels. However, the nanomaterials with lower density were adsorbed on the surface of biofilms, which interfered with the normal transportation of nutrients to some extent. Nanomaterials produced reactive oxygen species on the cell surface, which could also cause peroxidative damage and change biofilm permeability [33]. In addition, the EPS excreted during the biofilm development barrier against the reactive oxygen species [34]. Thus, the nanomaterials could accelerate cell growth and improve the morphological structure of the biofilms.

Figure 4D shows that the surface of the biofilms was surrounded by polymers, which were polysaccharides and glycoproteins outside the bacterial community. The presence of an internal gap between bacterial communities was the pathway through which bacteria acquired nutrients and excreted metabolic waste. Moreover, it could be seen that there was a wide heterogeneity in the structure of the biofilms, and there were significant differences in the size and metabolic activity of the bacteria in the deep and shallow areas.

Figure 4E shows that the structure of the biofilms was loose, because the graphene was adsorbed in the biofilm structure. The biofilm structure in the single-phase flow was a columnar structure (Figure S1), while the biofilm was flat and compact under segmented flow (Figure 4D). This was because of the influence of the fluid force generated by the aqueous phase on the biofilm. In addition, the air segments in the microchannel could lead to the compact biofilm. In the single-phase flow system, the high shear stress produced by the turbulent flow compresses the biofilm into a columnar shape. In the segmented flow system, the fluid force of the water phase restricts the transfer of oxygen and nutrients to the biofilms, thereby affecting the growth and development of the biofilms.

### 2.5. Characterization of Biofilm

**Figure 5:**
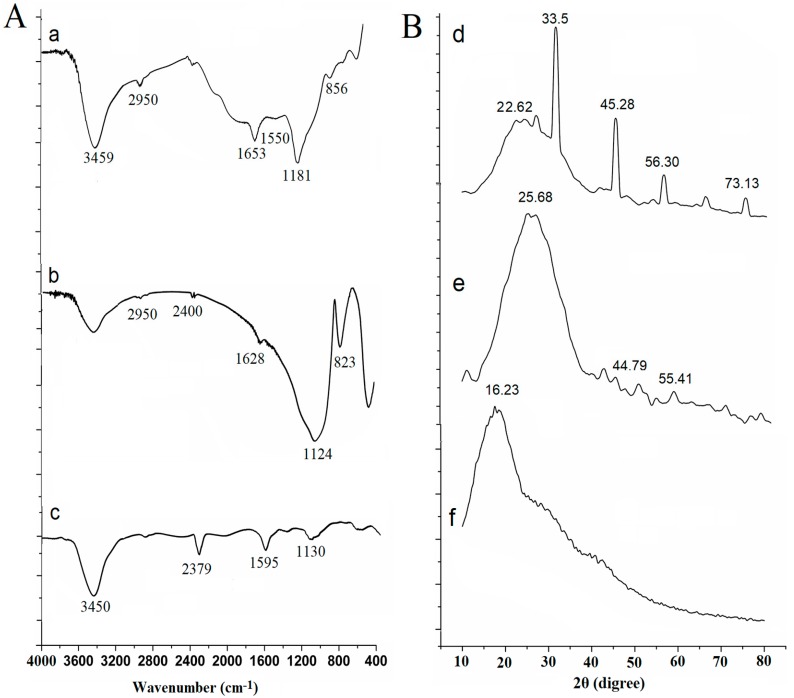
The FT-IR spectra (**A**) and X-ray diffraction patterns (**B**) of biofilms formed with LB medium and LB medium containing graphene nanomaterials (10 mg/L) in a microreactor by segment flow. Biofilm formed with LB medium (**a**,**d**); biofilm formed with LB medium containing graphene nanomaterials (10 mg/L) (**b**,**e**); graphene nanomaterials (**c**,**f**).

Figure 5A shows the FT-IR spectra of the biofilms formed with LB medium and LB medium containing graphene nanomaterials in microreactors by segment flow and graphene nanomaterials. In Figure 5A(a), the broad peak appearing at 3459 cm^−1^ was the result of an intramolecular or intermolecular O–H stretching vibration. The absorption peak at 2950 cm^−1^ was the result of C–H stretching vibration and bending vibration. The absorption peaks near 1653 cm^−1^ and 1550 cm^−1^ were amides I and II caused by N–H stretching vibration, which indicated that proteins are one of the components of EPS. The absorption peak of 1181 cm^−1^ was caused by C–O stretching vibration, which was caused by the C–O–H of ring breathing by polysaccharides and the C–O–C bond. 856 cm^−1^, shows the α-glycosidic bond of the polysaccharide molecules. The results therefore confirm the structures of polysaccharides and nucleic acids.

Figure 5A(c) shows that strong absorption peaks at about 3450 cm^−1^, 2379 cm^−1^, and 1595 cm^−1^ were from graphene [35]. Figure 5A(b) shows the biofilm formed with graphene nanomaterials. The absorption peak of graphene at 1595 cm^−1^ shifts to about 1628 cm^−1^, where the change of absorption peak is mainly caused by the stretching vibration of N–H and C=O of acetyl groups in the protein. At about 1124 cm^−1^, the absorption peak represented the P=O stretch present in nucleic acids [36]. The vibrational changes in this region might be caused by the inhibitory action of graphene nanomaterials during the formation of the biofilms. The absorption peak at 823 cm^−1^ shows the α-glycosidic bond of the polysaccharide molecules. Considering that the sample contained only the recombinant strain and the extracellular polymeric substance, it confirmed that the *E. coli* biofilm formed in the microreactor and graphene nanomaterials existed in the structure of the biofilms.

Figure 5B(d) shows that the recombinant *E. coli* biofilm in the segmented flow microchannel has diffraction peaks at 2θ = 22.62°, 2θ = 45.28°, 2θ = 56.30°, and 2θ = 73.13°. Using the software MDI Jade 6.5 analysis, the diffraction peak at 2θ = 22.62° represented galactose. The diffraction peaks at 2θ = 45.28° and 2θ = 73.13° attributed to D-glucose. The diffraction peak at 2θ = 56.30° resulted from glycine in the amino acid. The polysaccharides and amino acids represented by these peaks were important constituents of the biofilms. The high intensity of the diffraction peaks indicated that a large amount of extracellular polysaccharides and amino acid substances surround the recombination *E. coli*. These results suggested that recombination *E. coli* biofilms formed well in the microchannel reactor under the segmented flow mode.

From Figure 5B(e), the diffraction peak at 2θ = 25.68° represented the polysaccharide, and the diffraction peak at 2θ = 44.79° arose from glucosans. At 2θ = 55.41°, the diffraction peak was caused by amino acids. The intensity of the diffraction peaks was significantly decreased, compared with the peaks of biofilms cultivated with LB medium. This was because the graphene mixed with the EPS during the formation of biofilms. The results indicated that the presence of graphene nanomaterials affected the morphological structure of the biofilms.

In Figure 5B(f), the broad diffraction peak, at about 2θ = 16.23° observed in amorphous regions, was attributed to graphene sheets [37]. Compared with the graphene diffraction pattern, several diffraction peaks were added. This further confirmed that EPS joined the biofilm formation.

## 3. Materials and Methods

### 3.1. Materials

Glutaraldehyde and ATPES were purchased from Sigma Chemical Co., St. Louis, MO, USA. Nanometer materials were purchased from Dk Nano technology Co., Ltd., Beijing, China. All other essential solvents and reagents were of analytical grade. The flow-tube consisted of a capillary PFA tube with an inner diameter of 2 mm and a length of 130 mm.

### 3.2. Surface Modification and Immobilization of the Strain in the Microreactors

In this experiment, the microchannel was modified by using a modified protocol [15]. 98% ethanol solution was used to rinse the microchannel at a flow rate of 30 mL/min for 1 h. After that, the aqueous solution of APTES (1/9, *v*/*v*) was injected into the microchannel for 2 h at a flow rate of 30 μL/min. Then, glutaraldehyde-pure water (1/99, *v*/*v*) was injected for 30 min. Finally, the precultured recombinant *Escherichia coli* was introduced into the pipe and statically colonized for 2 h at 32 °C.

### 3.3. Recombinant E. coli Strains and Cultivation

The recombinant *E. coli* strain BL21-pET28a-*egfp* was inoculated in LB liquid medium containing 5 mL kanamycin (50 μg/mL) and incubated at 37 °C. The bacterial (1%, *v*/*v*) was transferred to LB liquid medium (500 mL) containing kanamycin (50 μg/mL), and cultured at 22 °C until OD600 reached 0.6 to 0.8. Then IPTG 400 μM was added and the temperature was lowered to 17 °C for 20 h to induce expression of egfp.

### 3.4. Growth of E. coli and Biofilm

The assembled microreactor was sterilized by autoclaving at 121 °C for 20 min. The PFA tube was first washed with 96% ethanol for 1 h. The PFA tube surface was then treated with freshly prepared 10% aqueous solution of APTES for 4 h, followed by treatment with 1% aqueous solution of glutaraldehyde for 30 min. *Escherichia coli* strains were applied to the flow tube and left to attach for 2 h. The LB medium was injected into PFA tube by a micropump with a flow rate of 45 μL/min for 60 h. Then air segments were introduced by another micropump at a flow rate of 45 μL/min. The culture system was operated in a segmented flow mode.

### 3.5. Total Biomass Assay of E. coli Biofilm

Crystal violet was used to assess formation of biofilms using a modified protocol [38]. Firstly, the air was used to blow to the culture medium by micropump. Then methanol was connected to micropump for a 20 min and the concentration of 0.1% crystal violet was used for biofilm dyeing, static staining for 30 min. After staining, the crystal violet was removed by PBS to remove the floating color and dried at room temperature for 30 min. Finally, 95% ethanol was used to dissolve the crystal violet adsorbed on the biofilm for 30 min. The total amount of biofilms was measured by optical density (OD) value at 590 nm.

### 3.6. Biofilm Cultivation by LB Culture Medium with Nanomaterials in Microchannels

The culture medium and different concentrations of carbon nanotubes, Fe_2_O_3_, and graphene nanomaterial suspensions (ultrasonic pretreatment for 30 min) were respectively mixed in Erlenmeyer flasks and sterilized at 121 °C for 20 min. The final concentration of nanomaterials in the mixture was 10, 20, 30, 40, and 50 mg/L, the mixed culture liquid after sterilization was used to culture the biofilms, and the fungal biomass of the microchannels was measured at different times. A blank control group was added with the same concentration of medium without nanomaterials. At the same time, the influence of the nanomaterials suspension on the absorbance value was excluded, and the medium containing different concentrations of nanomaterials was used as a blank control group. Samples were detected at 590 nm. The OD values were measured and the OD values of the blank control groups were finally subtracted.

### 3.7. Characterization Analysis

The prepared samples of biofilms were characterized by XRD patterns on a SHIMADZU XRD-6000X diffractometer system (Shimadzu Corporation, Nakagyo-ku, Kyoto, Japan). FT-IR spectra were recorded on a BRUKER TENSOR27 (Bruker Instruments, Ettlingen, Baden-Württemberg, Germany). The morphology of biofilms was studied by SEM (HITACHI S-4700, Hitachi Ltd., Tokyo, Japan). The morphology of the recombination strain immobilized on the surface of the microchannel was studied using a fluorescence inversion microscope system (Nikon ECLIPSE TS100, Nikon Instruments Europe B.V., Kingston, Surrey, England).

### 3.8. Statistical Analysis

Triplicate experiments were performed for each parameter investigated. Standard deviations were calculated to verify the reliability of the results. The differences in mean values were evaluated using the analysis of variance (ANOVA). Significance was determined at a 95% level of probability.

## 4. Conclusions

A microchannel reactor with surface modification was used to rapidly colonize *E. coli*, LB medium containing nanomaterials was used to cultivate biofilm by segmented flow. The experimental results showed that the optimum conditions were as follows. The optimal flow rate was 45 μL/min, the culture temperature was 32 °C, the optimum pH was 7.0, and the culture time was 36 h. The inhibition rates of graphene sheets, carbon nanotube, and Fe_2_O_3_ (10 mg/L) on the biofilm formation were 3.23%, 7.51%, and 16.13%, respectively. At the same concentration, the inhibitory effect of nanomaterials on biofilms was less than that of recombinant strains, which was attributed to EPS mitigating inhibition effects of nanoparticles during the formation of biofilms. The biofilm stability time was 20 h, which was 33.33% shorter than that by the LB medium without graphene. Thus, the developed method will provide an efficient and simple approach for improving the preparation efficiency of biofilm catalysts.

## Figures and Tables

**Table 1:** Effect of oxygen content on *E. coli* biofilm formation in the microchannel reactor.

Aqueous Flow Rate (μL/min)	Air Flower Rate (μL/min)	OD_590_
45	45	1.431 ± 0.052
45	55	1.492 ± 0.046
45	75	1.729 ± 0.049
45	95	1.828 ± 0.069
45	180	1.837 ± 0.051

**Table 2:** Different kinds of nanoparticles used in the present study.

Nanometer Materials	Diameter (nm)	Length (μm)	Density (g/cm^3^)
Short-multiwalled carbon nanotube	20–30	0.5–2	1.68
Fe_2_O_3_	-	30–50	5.18
Graphene	0.55–1.2	0.5–3	0.77

## Supplementary Materials

The following are available online at http://www.mdpi.com/1422-0067/19/9/2590/s1.
