# Supplementary material for: Recombinant Escherichia coli BL21-pET28a-egfp Cultivated with Nanomaterials in a Modified Microchannel for Biofilm Formation
Source: Int J Mol Sci. 2018 Aug 31;19(9):2590. doi: 10.3390/ijms19092590 (PMC6163294; doi:10.3390/ijms19092590)
Supplement: Supplementary file 1 [file ijms-19-02590-s001.pdf]

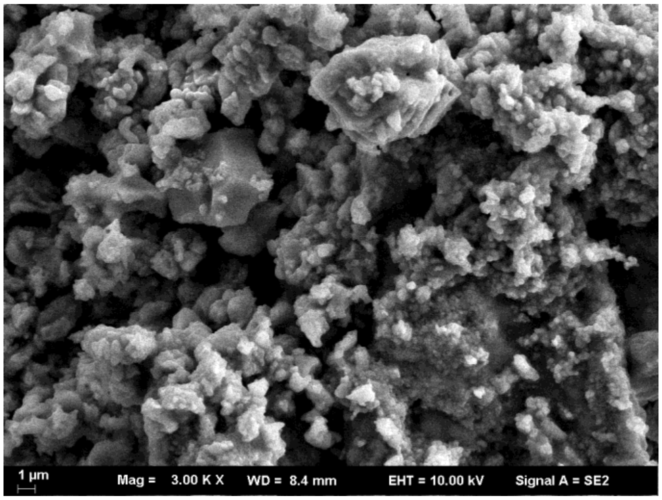

Figure S1. SEM photo of biofilms formed with LB medium in a microchannel reactor under single phase flow mode.
